# Polycaprolactone/Gelatin/Hyaluronic Acid Electrospun Scaffolds to Mimic Glioblastoma Extracellular Matrix

**DOI:** 10.3390/ma13112661

**Published:** 2020-06-11

**Authors:** Semra Unal, Sema Arslan, Betul Karademir Yilmaz, Faik Nuzhet Oktar, Denisa Ficai, Anton Ficai, Oguzhan Gunduz

**Affiliations:** 1 Department of Bioengineering, Faculty of Engineering, Marmara University, 34722 Istanbul, Turkey; unalsemra@gmail.com (S.U.); foktar@marmara.edu.tr (F.N.O.); 2 Center for Nanotechnology & Biomaterials Application and Research, Marmara University, 34722 Istanbul, Turkey; betulkarademir@marmara.edu.tr; 3 Institute of Neurological Sciences, Marmara University, 34722 Istanbul, Turkey; 4 Department of Biochemistry, School of Medicine/Genetic and Metabolic Diseases Research and Investigation Center, Marmara University, 34722 Istanbul, Turkey; semarsln1@hotmail.com; 5 Faculty of Applied Chemistry and Materials Science, University POLITEHNICA of Bucharest, 1-7 Gh. Polizu st, 060042 Bucharest, Romania; denisaficai@yahoo.ro; 6 Academy of Romanian Scientists, 3 Ilfov st. District 3, 050094 Bucharest, Romania; 7 Department of Metallurgy and Materials Engineering, Faculty of Technology, Marmara University, 34722 Istanbul, Turkey

**Keywords:** 3D extracellular matrix, glioblastoma tumor model, polycaprolactone, gelatin, hyaluronic acid, nanofiber

## Abstract

Glioblastoma (GBM), one of the most malignant types of human brain tumor, is resistant to conventional treatments and is associated with poor survival. Since the 3D extracellular matrix (ECM) of GBM microenvironment plays a significant role on the tumor behavior, the engineering of the ECM will help us to get more information on the tumor behavior and to define novel therapeutic strategies. In this study, polycaprolactone (PCL)/gelatin(Gel)/hyaluronic acid(HA) composite scaffolds with aligned and randomly oriented nanofibers were successfully fabricated by electrospinning for mimicking the extracellular matrix of GBM tumor. We investigated the effect of nanotopography and components of fibers on the mechanical, morphological, and hydrophilic properties of electrospun nanofiber as well as their biocompatibility properties. Fourier transform infrared spectroscopy (FTIR) and differential scanning calorimetry (DSC) have been used to investigate possible interactions between components. The mean fiber diameter in the nanofiber matrix was increased with the presence of HA at low collector rotation speed. Moreover, the rotational velocity of the collector affected the fiber diameters as well as their homogenous distribution. Water contact angle measurements confirmed that hyaluronic acid-incorporated aligned nanofibers were more hydrophilic than that of random nanofibers. In addition, PCL/Gel/HA nanofibrous scaffold (7.9 MPa) exhibited a significant decrease in tensile strength compared to PCL/Gel nanofibrous mat (19.2 MPa). In-vitro biocompatibilities of nanofiber scaffolds were tested with glioblastoma cells (U251), and the PCL/Gel/HA scaffolds with random nanofiber showed improved cell adhesion and proliferation. On the other hand, PCL/Gel/HA scaffolds with aligned nanofiber were found suitable for enhancing axon growth and elongation supporting intracellular communication. Based on these results, PCL/Gel/HA composite scaffolds are excellent candidates as a biomimetic matrix for GBM and the study of the tumor.

## 1. Introduction

Glioblastoma (GBM) is a highly malignant human primary brain tumor. Even with maximal surgical resection followed by adjuvant chemo-radiotherapy, median survival is still less than two years [1]. It is now well known that a physical component of tumor extracellular matrix (ECM) plays a crucial role in the ability of tumor progression, stiffness, and response to treatment strategies [2,3,4]. Thus, instead of the cell monolayer culture in the petri dish, the use of 3D scaffolds that mimic the ECM of the tumor can provide several advantages, such as mirroring the environment in which tumor cells live in the body, better replicating complex tissue structures. In addition, it reflects normal differentiation, cell behavior, and intercellular interactions; shows more realistic cell biology and function; and provides more accurate predictions of the disease states and drug response.

To date, natural and synthetic polymers were used to produce 3D scaffolds by various fabrication techniques. Electrospinning is a simple and commonly used technique for constructing 3D scaffolds consisting of nanofibers or mostly sub-microfibers-structured scaffolds [5,6,7] that closely mimic the dimensions of collagen fibril of GBM ECM [8,9]. In addition to fiber morphology, surface chemistry, mechanical properties, and cell–cell and cell–matrix interactions play a significant role for proper tissue engineering scaffold. A pure polymer is not adequate to obtain that required features to the scaffold; on the other hand, it is possible to adopt a hybrid scaffold consisting of the desired properties by using a polymer mixture. Polycaprolactone (PCL) is a synthetic polymer that was commonly used in biomedical applications due to its high biocompatibility, slow biodegradability, and good mechanical strength [10,11]. However, PCL scaffold needs surface modification to more accurately mimic ECM topography of glioblastoma multiforme (GBM) because its high hydrophobic surface chemistry leads to a low level circulation of biological fluids as well as proteins that induce cell adhesion, cell growth, migration and differentiation [12]. Gelatin, natural polymer denatured from collagen, which is a major component of ECM, has been extensively studied in tissue engineering applications due to its excellent properties, such as biocompatibility and biodegradability [13]. Gelatin is also non-immunogenic and has many integrin-binding sites, which promotes cell adhesion, differentiation, and proliferation [14]. Besides its advantages, its rapid degradation and poor mechanical properties are the main disadvantages of the use of gelatin in tissue engineering [14]. Thus, by combining gelatin with PCL, a composite scaffold having good mechanical strength, enhanced cell adhesion, and cell growth properties can be fabricated [15,16,17].

Another natural anionic polymer using in tissue engineering is hyaluronic acid (HA), which is found in skin and cartilage, and is an essential component of brain ECM [15]. Since the mechanical properties of pure hyaluronic acid scaffolds cannot always satisfy the clinical requirements, natural and synthetic polymers have been mixed with hyaluronic acid to produce composite scaffolds. For example, gelatin/hyaluronic acid composite scaffolds were fabricated using crosslinking [18,19,20,21] and electrospinning [22] strategies. Furthermore, hyaluronic acid was combined with polycaprolactone (PCL), a synthetic polymer commonly used for tissue engineering, to improve the mechanical properties of composite scaffolds. The porous composite scaffolds of PCL/hyaluronic acid [23,24] or PCL/gelatin [11,13,25,26,27,28,29,30,31,32,33,34,35,36] were manufactured by electrospinning. Thus, by combining the gelatin or HA with PCL, a composite scaffold with good mechanical strength, enhanced cell adhesion, and cell growth properties can be fabricated [15,16,17]. It is well known to be related with the growth and invasion of the GBM tumor, which is characterized by increased HA expression in the brain tumor stroma, and progressive margin [15,16,17,37,38,39]. Despite its biophysical properties, negative charge prevents cell adhesion [14]; thus, it is mixed with cationic biopolymers, including chitosan, gelatin, collagen to promote cell attachment [17,21,40,41,42]. Nano-sized fibers could mimic the natural extracellular architecture, and the addition of natural components to them may reveal structures that are more similar to the GBM extracellular environment. There are many studies utilizing PCL/gelatin nanofibrous scaffolds for various tissue engineering applications, including skin, nerve, bone, and vascular. However, there is no report PCL/Gel/HA composite nanofibrous scaffold in tissue engineering applications or even in 3D cell cultures.

The fiber diameter remained in the nanoscale range for all the studied scaffolds, which is crucial to mimic the extra-cellular matrix size, an essential requirement for satisfactory cell attachment and proliferation [22]. Rao et al. [9] produced aligned nanofiber with various shell materials (hyaluronic acid, collagen, and Matrigel) on PCL core. They reported that the nanofiber diameter ranged from an average of 800 to 900 nm for the scaffold. On the other hand, we demonstrated that the fiber diameter obtained from electrospinning of the blended solution of PCL and gelatin as well as the PCL/Gel electrospun nanofiber in the form of core-shell was lower to that found by other authors [24,33,34]. Unlike many studies on cellular responses of GBM to scaffold materials, we have examined the effect not only on nanotopography (random or aligned fiber morphology), but also on the components (gelatin, hyaluronic acid) contained in nanofibers. In this study, electrospinning of a blended solution of PCL, gelatin, and hyaluronic acid that has not been presented in the literature yet, was found to be an efficient technique to modify PCL nanofibrous scaffolds for 3D glioblastoma cell culture. Aligned and random PCL/Gel nanofiber scaffolds were produced for investigating the effects of nanotopography and hyaluronic acid addition in terms of surface chemistry, morphology, mechanical, and 3D cell culture applications. The cell growth and cell–matrix interaction of glioblastoma (U251) cells cultured on the scaffolds were evaluated in vitro, using tissue culture plastic (TCP) plates as controls.

## 2. Materials and Methods

### 2.1. Fabrications of Nanofiber Scaffolds

**Figure 1:**
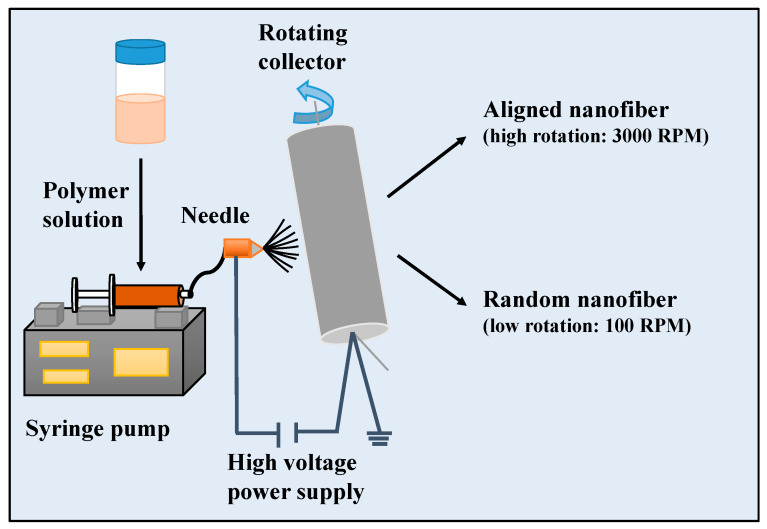
Schematic illustration for the fabrication of 3D PCL/Gel and PCL/Gel/HA0.5 composite scaffolds using electrospinning.

As shown in Figure 1, briefly,15 wt.% PCL and15 wt.% Gel polymers were separately dissolved in a formic acid/acetic acid solution (1:1 *v*/*v*). To obtain the PCL/Gel mixture solution containing 9 wt % PCL and 6 wt % Gel, the prepared polymer solutions were blended in 3:2 (*v*/*v*) ratio. HA was added to the obtained PCL/Gel polymer solutions at 0.5 wt % concentration. The homogeneous solutions were prepared using a magnetic stirring overnight at room temperature. Electrospinning setup consists of a polymer mixture placed in 10 mL syringe with an 18-gauge needle and rotating collector. Composite nanofibers were fabricated for an optimum condition: voltage of 22–25 kV, flow rate of 200–250 μL/h, 13 cm distance between needle and collector. The collector was rotated with the rotation speed of 3000 rpm for producing aligned fibers or 100 rpm for fabricating random fibers. After electrospinning, each sample was stored at ambient for 24 h before characterization. The obtained random nanofiber scaffolds were named as PCL/Gel, PCL/Gel/HA, while aligned nanofiber scaffolds were named as PCL/Gelxy and PCL/Gel/HAxy.

### 2.2. Measurement of Electrospinning Suspension

The conductivity, viscosity, and surface tension of prepared solutions were characterized at ambient temperature using an electroconductive meter (Cond 3110 SET 1 WTW, 82362 Weilheim, Germany), digital viscometer (DV-E, Brook- field AMETEK, USA), and a surface tension meter (the du Nouy procedure with a platinum ring), respectively. Each measurement was performed three times; the average value and standard deviation were calculated.

### 2.3. Characterization of Scaffolds

Scanning electron microscopy was used to determine the scaffold morphology (SEM, EVO MA-10, Zeiss, Germany). Fiber diameters were measured from randomly selected 100 fibers from SEM images (taken at 2000× magnification) with Image J software.

The composite scaffolds were characterized by Fourier transform infrared spectrometer (FTIR) (model 4600 Jasco, Japan).

Tensile properties of the scaffold were evaluated using a tensile test machine (EZ-LX model, Shimadzu, Japan) to obtain tensile stress–strain curves for all samples. To analyze the tensile properties of the aligned scaffolds, the lengths of the samples were cut both along the orientation of fibers (PCL/Gelx and PCL/Gel/HAx) and perpendicular to the orientation of fibers (PCL/Gely and PCL/Gel/HAy). The samples were cut into 5 cm (length) × 1 cm (width). Young’s modulus and ultimate tensile strength values were calculated through these curves.

For differential scanning calorimetry (DSC) analysis, samples (≅4 mg) were heated from 30 °C to 200 °C with a speed of a 5 °C/min and carried out on a DSC-60 Plus (DSC-60 Plus Thermal Analysis Instruments, Shimadzu, Japan).

The wettability of the scaffolds was characterized by water contact angles (θ) (WCA) measurements using the sessile drop method (drop shape analysis system DSA-100/10, Kruüss) at ambient temperature. Water droplets (3 μL of deionized water) were automatically dropped on the sample surface, and its evolution after 2 s was recorded with a CCD camera connected to the equipment. Then the equipment software automatically measured the WCA.

### 2.4. Cell Culture Study

Cell culture on the scaffolds—U251 cells were used for all cell experiments in Dulbecco’s modified eagle’s medium (DMEM) supplemented with 10% fetal bovine serum (FBS) and 1% penicillin/streptomycin. The samples with circle pieces (0.02 mm in thickness and 16 mm in diameter) were placed into 24-well plates, followed by ultraviolet (UV) light sterilization for 2 h. A density of 1 × 10^5^ cells/well was cultured on scaffolds and incubated at 37 °C in 5% CO_2_ incubator.

Viability—A *3*-(4,5-dimethylthiazol-2-yl)-*2*,5-diphenyl tetrazolium bromide (MTT) assay was used for the evaluation of cell viability. Cells were seeded on scaffolds as described above. After 1, 4, and 7 days of cell incubation, the cell-cultured scaffolds were washed with PBS and then incubated with 0.5 mL of 0.5 mg/mL MTT in PBS for 4 h at 37 °C with 5% CO_2_. The supernatant was removed gently, followed by the addition of 1.5 mL of dimethylsulfoxide (DMSO). Then, each sample was transferred to a 96-well plate to measure the absorbance at 590 nm (reference 660 nm) wavelength using a microplate reader.

Cell morphology—The cell–matrix interaction and cell morphology on scaffolds were evaluated by scanning electron microscope (SEM) and confocal laser scanning microscope. After 4 and 7 days of cell incubation, cell-laden scaffolds were fixed with 2.5% glutaraldehyde (Sigma) for 1 h and then dehydrated via ethanol dilutions (30%, 50%, 70%, 90%, and 99%) and dried in air. All samples were coated with gold and analyzed using SEM (EVO MA-10, Zeiss, Germany). For cell observation under a confocal laser scanning microscope (Zeiss LSM700), on day 7, the samples were washed with PBS, fixed with 4% formaldehyde for 1 h. Permeabilization was then performed with 0.1% Triton X-100 in PBS for 15 min, followed by washing with PBS. The samples were incubated with FITC–phalloidin (diluted 1:100) for 1 h at room temperature, followed by staining with DAPI. Confocal laser scanning microscope was used to obtain cell fluorescence images (20× immersion objective). The emission wavelengths were fixed at 500–540 nm for FITC and 461 nm for DAPI.

## 3. Results and Discussion

**Figure 2:**
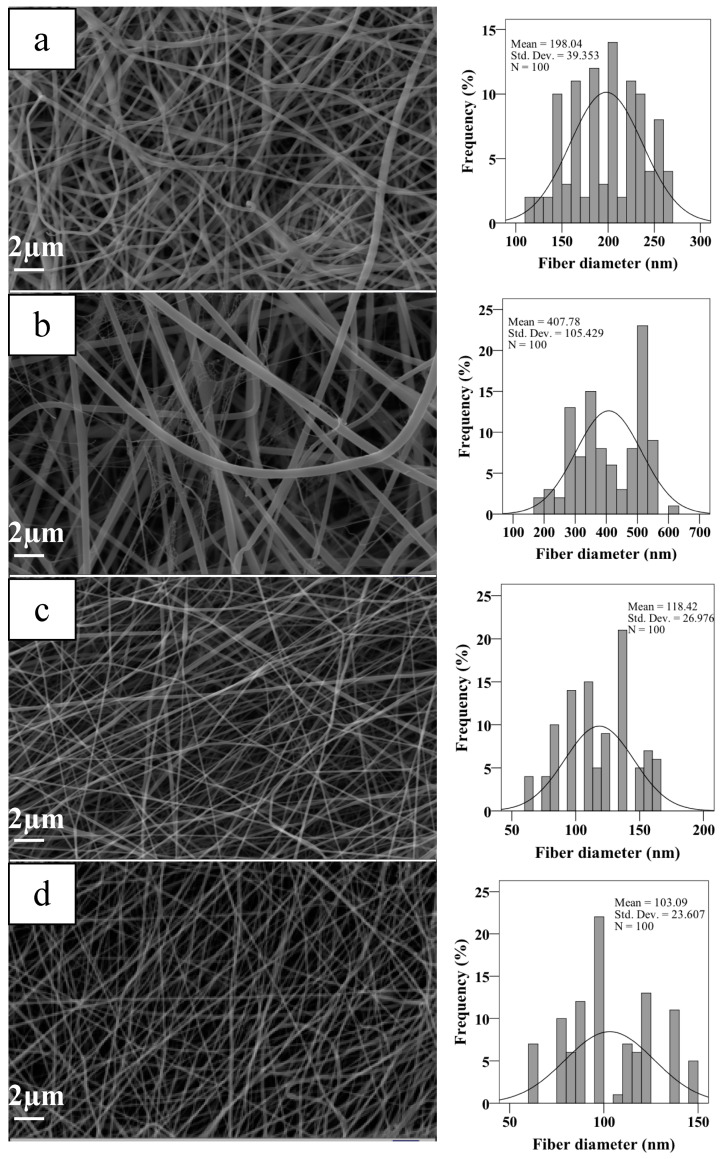
SEM images and fiber diameter distributions of scaffolds (**a**) PCL/Gel, (**b**) PCL/Gel/HA0.5, (**c**) PCL/Gelxy, and (**d**) PCL/Gel/HAxy.

As shown in Figure 1, aligned, and random nanofiber scaffolds were successfully fabricated with two-collector speed processing conditions applied in the electrospinning method. The optimum conditions for the fabrication of electrospun scaffolds were set as follows: applied voltage of 22–25 kV, and a flow rate of 200–250 μL/h. The effects of various parameters on the fiber morphology were discussed here, including the addition of HA in the PCL/Gel and collector rotation speed. The PCL/Gel nanofiber diameter obtained from the electrospinning of the mixture solution of formic/acetic acid was found lower to that fabricated by other studies that used conventional halogenated solvents [14]. The solvent properties such as surface tension, viscosity, and conductivity have a significant effect on nanofiber morphology. Therefore, these were characterized and reported in Table 1. The surface tension and viscosity of PCL/Gel solution was slightly increased compared to the PCL/Gel/HA0.5 solutions, as shown in Table 1. Figure 2 represented nanofiber morphology and diameter distribution of nanofibers of PCL/Gel, PCL/Gel/HA, and PCL/Gelxy, PCL/Gel/HAxy fabricated at low and high-speed collector rotation processing, respectively. It can be revealed that fibers showed smooth morphology in all cases. The average nanofiber diameter varied depending on whether the nanofibers were aligned or randomly oriented. The fiber diameter increased from 198.04 ± 39.3 nm to 407.78 ± 105.4 nm with the addition of HA in the PCL/Gel. In this case, fiber distribution altered from uniform formation to non-homogeneous with the presence of HA in the PCL/Gel scaffold. The diameter of fibers produced at high-speed collector rotation decreased from 118.42 ± 26.9 nm to 103.09 ± 23.6 nm with the presence of HA in the PCL/Gelxy. Regardless of the hyaluronic acid content, the fiber diameter was significantly reduced for aligned nanofibers compared to random nanofibers. Similar results have been reported by Gil-Castell [34]. Compared to our results, they found a fiber diameter similar to the randomly oriented nanofibers but larger than the aligned nanofibers. At low collector rotation speed, it can be seen that randomly oriented smooth fibers with non-homogeneous diameter distribution were obtained, and the average diameter was observed to increase almost three times with the addition of HA in the spinning solution (Figure 2). This could be explained by the increased viscosity of the electrospinning solution due to the addition of hyaluronic acid. Similar results on the effect of viscosity on fiber diameter have been reported by Ke [36]. The result showed that the conductivity increased with the presence of HA content. HA is a polyelectrolyte, and the addition of HA to PCL/Gel solution could result in an increase in ion strength, which might result in higher conductivity. Since PCL is a non-ionic synthetic polymer, it does not produce ions in solution when dissolved in an organic solvent (formic acid/acetic acid; 1:1 *v*/*v*), on the other hand, gelatin, a natural polyelectrolyte, has several ionizable amino groups and carboxylic groups and can be generally hydrolyzed by either acidic condition or at neutral pH [14]. It was observed that the aligned nanofibers have a thinner nanofiber diameter than random nanofibers. The reason behind this result may be due to the difference in the electrical field distribution and the stretching force caused by the processing conditions [43].

**Figure 3:**
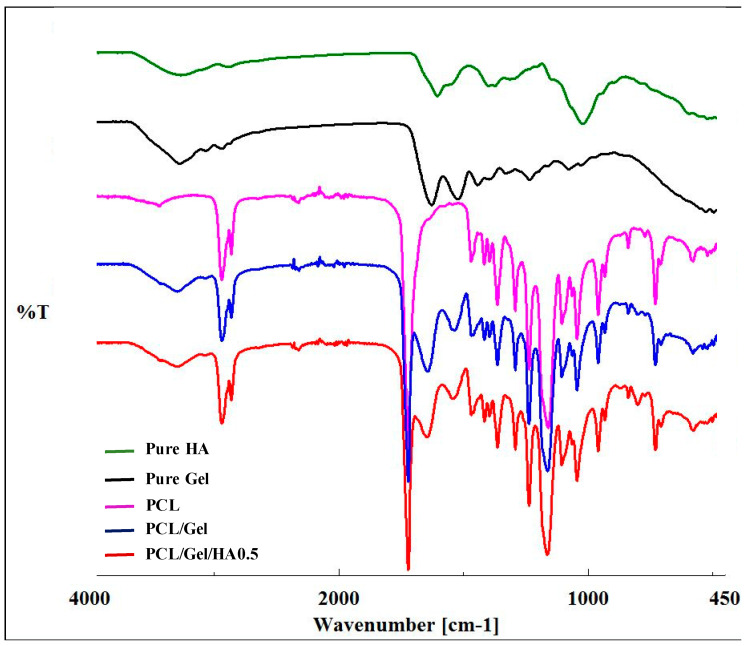
FTIR spectra of HA, Gel, PCL, PCL/Gel, and PCL/Gel/HA scaffolds.

FTIR spectroscopy was used to determine the molecular structure of HA, Gel, PCL, PCL/Gel, and PCL/Gel/HA0.5. PCL-related infrared spectra were observed in the curves of PCL, PCL/Gel, and PCL/Gel/HA0.5 scaffolds, as shown in Figure 3. The spectrum of PCL revealed several characteristic peaks, which were 1240 cm^−1^ and 1170 cm^−1^ associated with CH_2_ asymmetric and symmetric stretching vibrations, respectively; 1727 cm^−1^ indicated C=O stretching vibration in carbonyl groups; 2950 cm^−1^ and 2865 cm^−1^ corresponding to C–O–C asymmetric and symmetric stretching vibrations, respectively [26,44]. Gelatin-related characteristic bands exhibited at about 1650 cm^−1^, 1540 cm^−1^, and 3300 cm^−1^, which were associated with C=O stretching vibration, the coupling of the N—H in plane bending and the C—N stretching vibration, and the N—H stretching vibration, respectively [26,35,45]. For HA, the typical bands were found at about 3283 cm^−1^, 1044 cm^−1^, and 1614 cm^−1^ which were indicated to the hydrogen-bonded O–H stretching vibration, the C–O–C stretching vibration and the carbonyl group of the carboxylate (COO–) asymmetric stretching vibration, respectively [15,23]. According to Xue et al., by the presence of acetic acid, the chain entanglement and hydrogen interaction between PCL and gelatin increase [29]. FTIR spectra confirmed the shifting of the position at amide I towards a higher wavenumber in PCL/gelatin and PCL/Gel/HA composite scaffolds. Many researchers indicate that there might be a hydrogen-bonding interaction between the ester group of PCL and amine group of gelatin molecules within the scaffold [14,29,33,46]. The entire characteristic peaks of PCL, gelatin and HA were detected in the spectra of the PCL/Gel/HA scaffolds, indicating the presence of the polymers in the composite scaffolds.

**Figure 4:**
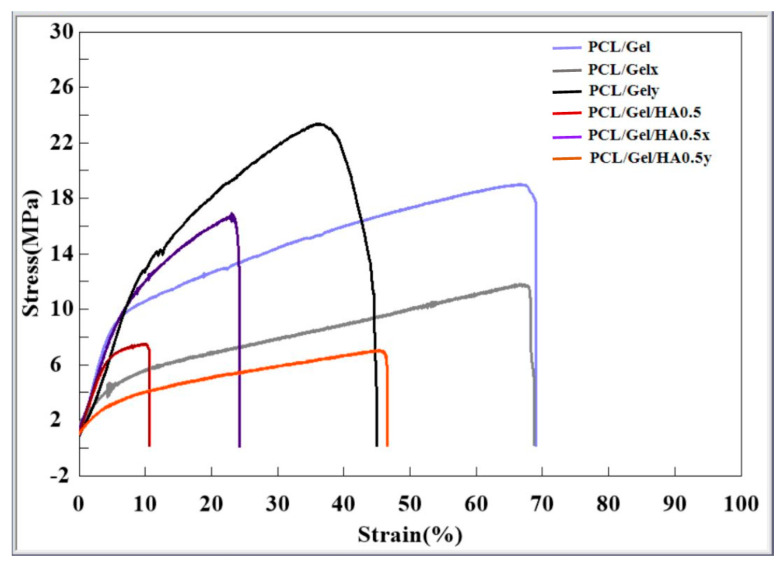
Stress–strain curves of PCL/Gel, PCL/Gelxy, PCL/Gel/HA, and PCL/Gel/HAxy scaffolds.

The stress–strain properties of scaffolds fabricated with two spinning parameters, including low and high collector rotation speed were characterized at 25 °C. The typical stress–strain curves are presented in Figure 4. Elongation at break of aligned PCL/Gel/HA fibers was measured 69%, while those of random fibers decreased to 11%. On the other hand, there is no significant change in the elongation properties of between PCL/Gelxy and PCL/Gel/HAxy composite scaffolds. Also, tensile strength for PCL/Gel, PCL/Gelxy, PCL/Gel/HA, and PCL/Gel/HAxy scaffolds were 19.2 ± 0.9, 23 ± 2.1, 7.9 ± 0.8, and 7.1 ± 1.4 MPa, respectively. PCL/Gel also exhibited a significant increase in tensile strength compared with the PCL/Gel/HA fibers. The presence of HA into the PCL/Gel fibers revealed no significant change in tensile strength compared with the PCL/Gel/HAxy that were fabricated by high-speed collector rotation fibers tested at room temperature. There are few studies investigated in GBM extracellular matrix and its mechanical properties. Rao et al. reported that the high migration speed was observed on the core/shell PCL-based aligned fiber scaffold with the module at 8 MPa [24]. Therefore, it is seen that the PCL/Gel/HA0.5 composite is more suitable when it is evaluated in terms of the mechanical properties of the GBM extracellular matrix.

**Figure 5:**
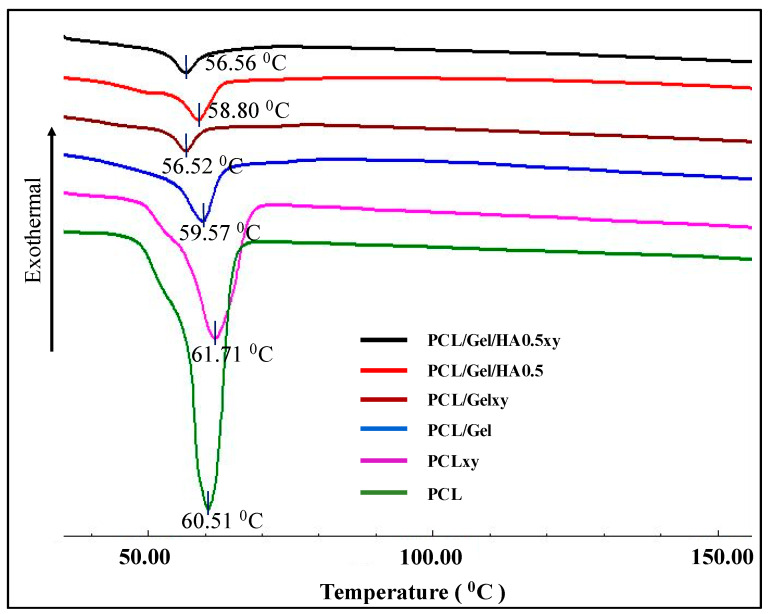
DSC thermograms of PCL, PCLxy, PCL/Gel, PCL/Gelxy, PCL/Gel/HA, and PCL/Gel/HAxy.

As shown in Figure 5, melting temperatures for PCL, PCLxy, PCL/Gel, PCL/Gelxy, PCL/Gel/HA, and PCL/Gel/HAxy scaffolds were 60.51, 61.71, 59.57, 56.52, 58.80, and 56.56 °C, respectively. With the Gel content of a nanofiber membrane, the endothermic melting peak of the PCL shifts toward a lower temperature. The melting points of PCL and PCLxy are 60.51 °C and 61.71 °C, respectively, while endothermic peaks in PCL/Gel and PCL/Gelxy scaffolds can be characterized as shifted to 59.57 °C and 56.52 °C, respectively. The reason for this might be attributed to water loss in the gelatin sample as a result of evaporation, which is similar to the literature [47]. The further decrease has been seen with the addition of HA to PCL/Gel composite scaffold. Sonseca et al. analyzed in melting temperature between random and aligned electrospun nanofibers. They showed that the melting temperature of aligned nanofibers is less than the one observed on random ones for some concentrations [48].

**Figure 6:**
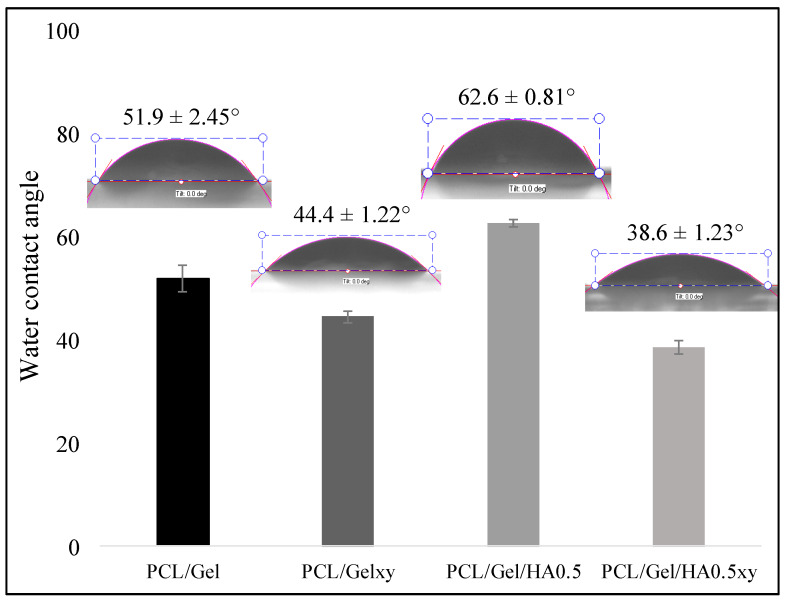
Water contact angle (WCA) of PCL/Gel, PCL/Gelxy, PCL/Gel/HA, and PCL/Gel/HAxy scaffolds.

The WCA measurement was used to evaluate the hydrophilic/hydrophobic properties of scaffolds which affect protein penetration and consequently cell adhesion and cell infiltration [12]. Studies in the literature have shown the WCA measurements of PCL nanofiber scaffolds ranging from 118° to 130°, demonstrating the hydrophobic property of PCL nanofiber scaffolds [12,29,35]. The addition of gelatin imparts a hydrophilic character to the PCL scaffold because of its amino and carboxyl functional groups [29]. The WCA test was performed to identify the influence caused by the incorporation of HA into PCL/Gel scaffold. The fiber nanotopography also plays a significant role in the nature of the wettability of scaffolds. The water contact angle values of the random and aligned nanofibers scaffold were evaluated, and the average angles were 51.9 ± 2.45° for PCL/Gel and 44.4 ± 1.22° for PCL/Gelxy, 62.6 ± 0.81° for PCL/Gel/HA, and 38.6 ± 1.23° for PCL/Gel/HAxy. As shown in Figure 6, the addition of HA significantly decreased the hydrophilicity of the random nanofibers scaffold, and the WCA changed from 51.9 ± 2.45° for PCL/Gel to 62.6 ± 0.81° for PCL/Gel/HA. On the other hand, the presence of HA showed a reverse effect on the aligned nanofibers scaffold, and the WCA decreased from 44.4 ± 1.22° for PCL/Gelxy to 38.6 ± 1.23° for PCL/Gel/HAxy. Previous research has demonstrated that surface chemistry of fibers could influence cell adhesion, spreading, and growth [23,49,50,51]. The hydrophilic/hydrophobic nature of scaffolds is very important in the interaction between the cells and the extracellular matrix [23,49,50,51]. Thus, the hydrophilic nature of PCL/Gel, PCL/Gelxy, PCL/Gel/HA0.5, and PCL/Gel/HA0.5xy composite scaffolds was determined to look to the surface water wettability, which influences nutrient diffusion, penetration of protein, and consequent cell adhesion and migration of cells into the scaffold [45,52,53]. The surface wettability of the aligned nanofibers was increased due to the increased porosity and its pore shape in the nanofiber [6]. This result shows that polymeric biomedical scaffolds with improved wettability can be fabricated with aligned fiber structure.

**Figure 7:**
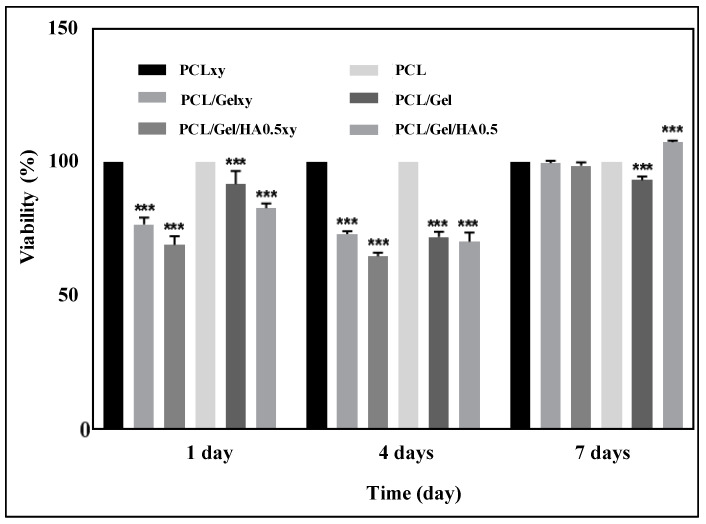
Cell viability of U251 cells cultured on PCL, PCLxy, PCL/Gel, PCL/Gelxy, PCL/Gel/HA, and PCL/Gel/HAxy scaffolds for 1, 4, and 7 days. Data are the means ± S.E.; n = 3; ***, *p* ≤ 0.001 versus control, ANOVA, Bonferroni’s multiple comparison test.

Cell survival, as well as cell distribution and morphology, is one of the parameters to be considered to evaluate the success of tissue engineering scaffolds [34]. In this study, U251 cells were cultured on scaffolds with random (PCL/Gel and PCL/Gel/HA0.5) and aligned (PCL/Gelxy and PCL/Gel/HA0.5xy) nanofiber orientations for 1, 4, and 7 days and biocompatibility tests of the scaffolds were performed using MTT assay, SEM, and confocal microscopy. The Figure 7 showed that after 1, 4, and 7 days of cell incubation, increased cell proliferation was demonstrated on the PCL/Gel/HA0.5 scaffold compared to the PCL/Gel/HA0.5xy and PCL/Gel and PCL/Gelxy scaffolds. Our results are consistent with those reported by many researchers, where they found that the cell viability incubated on PCL/Gel electrospun scaffolds showed good biocompatibility of nanofibers. For PCL/Gel scaffolds tested with various cell lines, cell viability was nearly over 80%; this indicates that all scaffolds are non-toxic and provided cell proliferation [11,14,34,36,47]. As described in the previous section, the PCL/Gel/HA0.5 composite scaffold exhibited favorable properties in terms of water contact angle and tensile strength, which strongly influence cell adhesion and proliferation behavior. In general, wettability properties of the scaffolds are essential in 3D cell culture and can significantly affect initial cell adhesion and cell infiltration into nanofibers [54]. Therefore, since the PCL/Gel/HA0.5 scaffold exhibited a moderate hydrophilic surface property (62.6 ± 0.81°), the growth rate of U251 cells may be increased.

**Figure 8:**
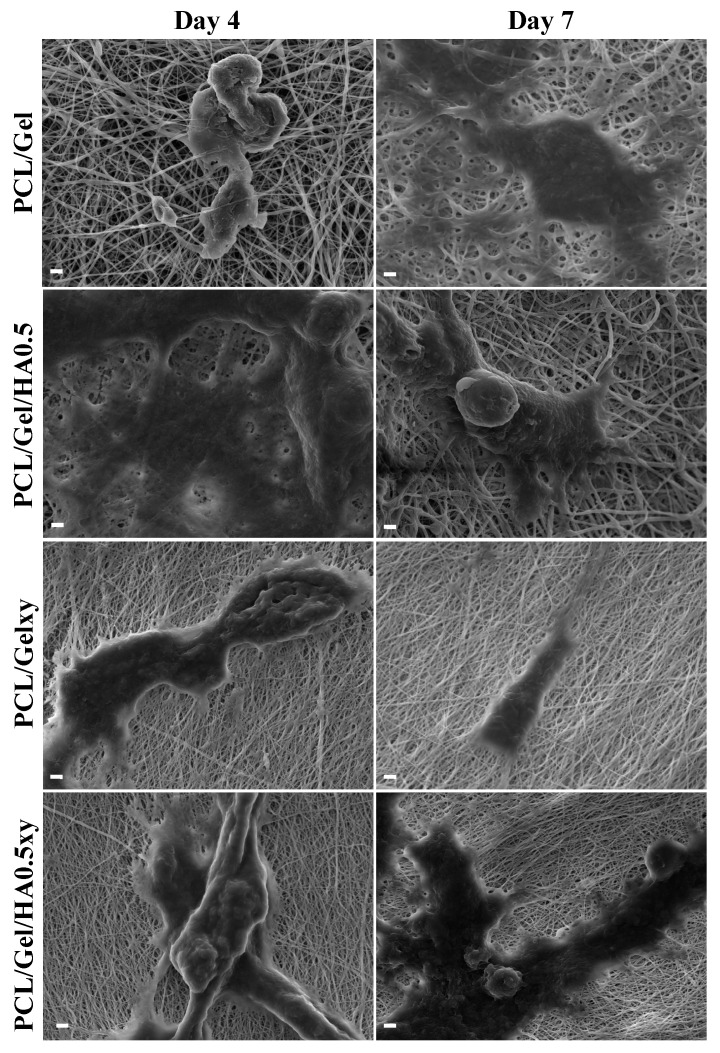
SEM images of U251 cells attached to scaffolds after 4 and 7 days of incubation (scale bar: 2 μm).

**Figure 9:**
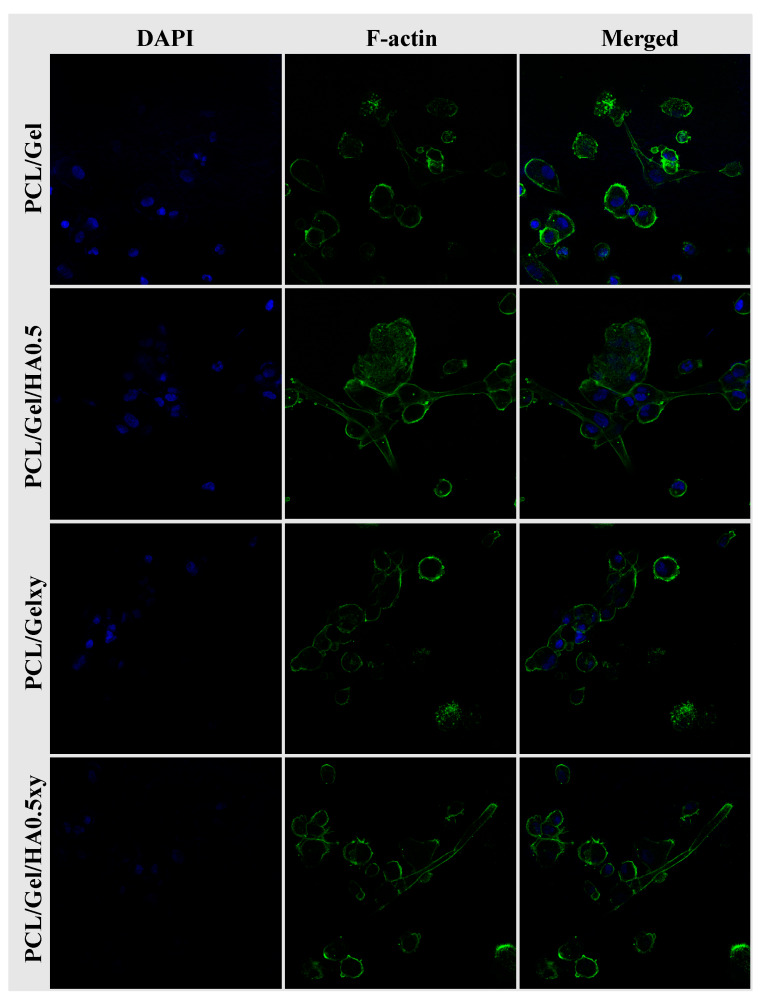
Confocal microscopy images of U251 cells seeded on scaffolds. Cells on scaffolds were stained with DAPI to display blue (nuclei) and F-actin to show green (cytoskeleton).

The SEM images in Figure 8 show that all scaffolds had excellent biocompatibility and promoted cell attachment, spread, and proliferation. While the cell attachment on the aligned nanofiber scaffolds tends to be a spindle-like shape through elongated filopodia formation, the cells seeded on the random nanofibers scaffold demonstrated a flattened morphology. The 3D culture, which mimics the characteristics of the GBM microenvironment, should include HA [15,16,17,54,55] but the amount of HA in scaffolds plays a vital role for this purpose due to its effect on the lack of mechanical strength and inhibiting cell adhesion because of its anionic structure [55]. Rao et al. showed that patient tumor derived OSU-2 cells seeded on Col-HA based 3D hydrogels showed a spindle-shaped morphology (we observed the similar result as mentioned above), better spreading and migration at low concentration of HA (i.e., ≤0.2 wt % HA) [40]. On the other hand, OSU-2 cells cultured in Col-HA based hydrogels containing increased HA concentration exhibited a round cellular morphology as well as lost the ability to pass through the gel structure [40]. We observed a positive correlation with the wettability and the initial growth rate of the U251 cells. However, even though the wettability of PCL/Gel/HA0.5xy (the aligned nanofibers) is more excellent than PCL/Gel/HA0.5 (the random nanofibers), after 4 and 7 days, there was a significant difference in the number of cells between HA-based electrospun scaffolds with random and aligned nanofibers, indicating that the cell growth on random nanofiber scaffolds after the initial cell adhesion was higher than that of aligned nanofiber scaffolds. As shown in Figure 9, confocal microscopy images also show the cell growth behavior and the extracellular matrix–cells interaction according to structure and component of the nanofibers over the scaffolds. Cell adhesion on PCL/Gel/HA0.5 scaffold surface was seen as a rounded-cell shape in compacted mass form. Also, several cells in the formation of clusters tend to spread and elongate as the length of the actins of the cells and have developed cell–cell communication with cells in other clusters. Actin green stained elongated actins were observed in some of the U251 cells cultured on HA containing aligned nanofibers matrix, while cells cultured on PCL/Gelxy were rounded shape. In general, the formation of clusters and cell shapes on PCL/Gel based electrospun scaffolds show that 3D nanofiber scaffolds provide a biomimetic microenvironment as compared to monolayer culture by allowing cells to produce more cell–cell and cell–ECM contact.

## 4. Conclusions

In this study, for the first time, composite nanofibrous scaffolds with random and aligned nanofiber consisting of polycaprolactone, gelatin, and hyaluronic acid were manufactured by electrospinning method, using a solvent mixture: formic acid/acetic acid. Fiber diameter and distribution affected by the addition of HA content into PCL/Gel solution and changing of spinning parameters. The aligned nanofiber production was achieved under the influence of two main forces, including electrostatic force and the stretching force and these forces resulted in a reduction of nanofiber diameter. PCL/Gel/HA nanofibrous scaffold (7.9 MPa) displayed a significant decrease in tensile strength compared to PCL/Gel nanofibrous mat (19.2 MPa). The presence of HA decreased the mechanical properties, but their nanotopography did not cause a significant difference. The aligned nanofibers with the presence of HA exhibited superhydrophilic properties. Still, the random nanofibers containing HA have the moderately hydrophilic characteristic that can assist in cell growth compared to aligned nanofibers. Biomaterial topography and surface chemistry played an important role in the biomechanical, proliferative, and morphological properties of GBM cells. Overall, aggregated cell formation and cell morphology on PCL/Gel/HA0.5 composite scaffold indicate that this 3D scaffold with random nanofibers can be a promising candidate for the biomimetic platform of GBM extracellular matrix.

## Figures and Tables

**Table 1:** Physical properties of the solutions used in the current study

Sample	Viscosity (Pa s)	Surface Tension (mN m^−1^)	Conductivity (μS cm^−1^)
PCL/Gel	2.86 ± 0.04	29.85 ± 0.2	813 ± 7
PCL/Gel/HA0.5	3.05 ± 0.01	30.44 ± 0.3	1100 ± 10
